# Leptin Receptor Gene Variant rs11804091 Is Associated with BMI and Insulin Resistance in Spanish Female Obese Children: A Case-Control Study

**DOI:** 10.3390/ijms18081690

**Published:** 2017-08-03

**Authors:** Josune Olza, Azahara I. Rupérez, Mercedes Gil-Campos, Rosaura Leis, Ramón Cañete, Rafael Tojo, Ángel Gil, Concepción M. Aguilera

**Affiliations:** 1 Department of Biochemistry and Molecular Biology II, Faculty of Pharmacy, Institute of Nutrition and Food Technology, University of Granada, Av. Del Conocimiento s/n., 18016 Granada, Spain; jolza@ugr.es (J.O.); azahararuperez@ugr.es (A.I.R.); agil@ugr.es (Á.G.); 2 CIBER Fisiopatología de la Obesidad y la Nutrición (CIBEROBN), Instituto de Salud Carlos III, 28029 Madrid, Spain; mercedes_gil_campos@yahoo.es (M.G.C.); mariarosaura.leis@usc.es (R.L.); em1caesr@uco.es (R.C.); 3 Instituto de Investigación Biosanitaria ibs.GRANADA, 18012 Granada, Spain; 4 Paediatric Research and Metabolism Unit, Reina Sofía University Hospital, Maimonides Institute for Biomedical Research (IMIBIC), Av. Menendez Pidal s/n., 14010 Córdoba, Spain; 5 Unit of Investigation in Nutrition, Growth and Human Development of Galicia, Paediatric Department, Clinic University Hospital of Santiago, University of Santiago de Compostela, Travesia de Choupana, 15706 Galicia, Spain rafael.tojo@usc.es

**Keywords:** *LEPR* gene, genetic polymorphism, obesity, child, insulin resistance

## Abstract

Leptin is an endocrine hormone that has a critical role in body weight homoeostasis and mediates its effects via the leptin receptor (LEPR). Common polymorphisms in the genes coding leptin receptors have been associated with metabolic abnormalities. We assessed the association of 28 *LEPR* polymorphisms with body mass index (BMI) and their relationship with obesity-related phenotypes, inflammation and cardiovascular disease risk biomarkers. A multicentre case-control study was conducted in 522 children (286 with obesity and 236 with normal-BMI). All anthropometric, metabolic factors and biomarkers were higher in children with obesity except apolipoprotein (Apo)-AI, cholesterol, high-density lipoprotein cholesterol (HDL-c), and adiponectin, which were lower in the obesity group; and glucose, low-density lipoprotein cholesterol (LDL-c), and matrix metalloproteinase-9 that did not differ between groups. We identified the associations between rs11208659, rs11804091, rs10157275, rs9436303 and rs1627238, and BMI in the whole population, as well as the association of rs11804091, rs10157275, and rs1327118 with BMI in the female group, although only the rs11804091 remained associated after Bonferroni correction (*p* = 0.038). This single nucleotide polymorphisms (SNP) was also associated with insulin (*p* = 0.004), homeostasis model assessment for insulin resistance (HOMA-IR) (*p* = 0.006), quantitative insulin sensitivity check index (QUICKI) (*p* = 0.005) and adiponectin (*p* = 0.046) after adjusting for age, Tanner stage and BMI. Our results show a sex-specific association between the rs11804091 and obesity suggesting an influence of this SNP on insulin resistance.

## 1. Introduction

Paediatric obesity is a complex condition originated from both environmental and genetic factors [1]. It has become a worldwide public health concern as it has dramatically increased over the past three decades [2,3]. Genetic association studies have provided insights into the genetics of early-onset obesity, identifying strongly associated genes with large phenotypic effects such as the leptin (*LEP*) or leptin receptor (*LEPR*) genes [4]. Leptin is an endocrine hormone mainly produced by the adipose tissue, which has a critical role in body weight homeostasis [5]; its circulating levels are correlated with the amount of body fat and reflect the nutritional status [6]. Leptin mediates its effects via the leptin receptor, which is a member of the Class I cytokine receptor family and consists of an extracellular ligand-binding transmembrane domain and a cytoplasmic signalling domain [7]. Once in the bloodstream, leptin reaches the brain by crossing the blood–brain barrier (BBB), and binds to its receptor in the hypothalamus arcuate nucleus; there, the activation of the receptor inhibits the action of orexigenic peptides and stimulates anorectic neuropeptides, ensuing in the control of appetite and food intake [8]. Leptin also participates in the regulation of glucose homeostasis and insulin sensitivity, since these two hormones exert opposite effects and regulate each other in such a way that leptin inhibits insulin, and insulin stimulates leptin synthesis and secretion [9].

Leptin resistance defines a state of obesity where hyperleptinemia or diminished responsiveness to this hormone is observed. The lack of response to leptin due to the development of resistance may disturb central and peripheral actions of this hormone. Although some proposals have been made such as failure of leptin crossing the BBB, inhibition of the leptin signalling cascade, or a decrease in the expression of leptin receptors, to date the mechanisms underlying leptin resistance remain unclear [10].

In both the *LEP* and *LEPR* genes, homozygous mutations have been described that derive in extreme obesity [11,12,13]. In addition, a variety of single nucleotide polymorphisms (SNPs) in different loci have been associated with circulating leptin levels and obesity [14,15,16]. In the *LEPR* gene, different SNPs conferring increased susceptibility to common forms of obesity have been identified in adults [16], families [16,17,18,19,20,21,22,23] and children [24,25,26,27,28,29,30] with different ethnic backgrounds. In addition, other variants such as Gln223Arg (rs1137101) in this gene have been associated with type 2 diabetes [31]. However, although the common forms of obesity are a major public health problem and the impairment of the action of leptin and its receptor are implicated in the onset of obesity, few studies have investigated the contribution of *LEPR* genetic variants to the susceptibility to childhood obesity also focusing on their association with circulating biomarkers.

With all this in mind, the present study was undertaken with the aim of elucidating the possible association regarding *LEPR* variants in the setting or development of obesity in children. For this, we examined the association of 28 *LEPR* polymorphisms with body mass index (BMI) and analysed their relationship with biomarkers of insulin resistance, inflammation and cardiovascular disease (CVD) risk in Spanish children.

## 2. Results

### 2.1. General Characteristics of the Population

Table 1 shows the anthropometric, clinical and metabolic characteristics of the studied groups, as previously published [32]. As expected, weight, height, BMI, BMI *z-Score* (*z*-BMI) and waist circumference (WC) were significantly higher in children with obesity compared to children with normal-BMI. Systolic and diastolic blood pressure (BP), as well as plasma triacylglycerols (TAG), apolipoprotein (Apo)-B, insulin, and homeostatic model assessment of insulin resistance (HOMA-IR), were higher in children with obesity, whereas the quantitative insulin sensitivity check index (QUICKI), plasma total cholesterol, high-density lipoprotein-cholesterol (HDL-c) and Apo-AI were lower in this group when compared with normal-weight children. Fasting plasma glucose and low-density lipoprotein-cholesterol (LDL-c) concentrations showed no differences between groups. The concentrations of alanine transaminase and γ-glutamyl transpeptidase were higher in the obesity group, while that of aspartate transaminase was lower.

Plasma leptin concentration was significantly higher in subjects with obesity than in the normal-BMI subjects, whereas adiponectin was lower. Inflammation and most of the CVD risk biomarkers differed between groups. C-reactive protein (CRP), interleukin (IL) 6, IL-8, and tumour necrosis factor alpha (TNF-α) were higher in the obesity group compared with the normal-BMI group. Likewise, plasma soluble intracellular adhesion molecule-1 (sICAM-1), soluble endothelial selectin (sE-selectin), myeloperoxidase (MPO) and active and total plasminogen activator inhibitor (PAI-1) were higher in children with obesity, whereas matrix metalloproteinase-9 (MMP-9) showed no differences between groups.

### 2.2. Association of LEPR SNPs with Obesity

Among the 28 analysed SNPs, only the rs11208659, rs11804091, rs10157275, rs9436303, and rs1627238 were significantly associated with obesity in children, after age, sex and Tanner stage adjustment under an additive model (Table 2). However, none of the SNPs remained significantly associated after Bonferroni correction. When we performed the analysis separately by sex, we observed that rs11804091 and rs10157275, and additionally, rs1327118, were associated with BMI only in the female group, although only rs11804091 remained statistically significant after Bonferroni correction (OR = 2.73 for allelic effect, 95% CI: 1.47–5.08, *p* = 0.038). No association was observed between these SNPs and obesity in the male group; only rs11208659 showed a negative association with obesity in males that was lost after Bonferroni correction (Table 3). However, the previously described in adults SNPs, rs1137101, rs1137100, and rs8179183, were not associated with BMI in our population. Additionally, haplotype analyses showed that none of the significantly associated SNPs were in linkage disequilibrium (LD) with the three mentioned above, either considering the whole population or when analyses were performed separately by sex (Figures S1–S3).

Since our population came from two different cities of Spain, we performed a meta-analysis to avoid population stratification biases or a genotyping batch effect. The results of this analysis (*p* values of *Q* Cochrane: (rs11208659, *Q* = 0.997; rs11804091, *Q* = 0.228; rs10157275, *Q* = 0.304; rs9436303, *Q* = 0.166; and rs1627238, *Q* = 0.887) indicates little detectable heterogeneity for the two considered populations of the study.

To investigate the potential functional role of these variants, different web-based tools designed for in silico prediction of SNP function were queried using the SNP IDs, including the FuncPRED tool of the National Institutes of Health [33], RegulomeDB [34], MirSNP [35], and RegSNP [36]. The search showed no determinant effect of the significantly obesity associated SNPs on the binding of known transcription factors or microRNAs. Moreover, other variants found to be in LD with the associated SNPs, including one missense polymorphism, were not predicted to influence the function of the protein or the binding of transcription factors or microRNAs to the *LEPR* gene.

### 2.3. Association of SNP rs11804091 with Obesity-Related Traits

Table 4 shows the association of rs11804091 in the female population with anthropometric, clinical, inflammation and CVD risk markers adjusted by age and Tanner stage. This SNP was significantly positively associated with weight, *z*-BMI, systolic BP, insulin, HOMA-IR, Apo-AI, leptin, and TNF-α; and significantly negatively associated with height, QUICKI and adiponectin. This negative association with height could be due to the lower mean age of the GG genotype group (7.9 years). After an additional adjustment for BMI, insulin, HOMA-IR, QUICKI and adiponectin remained significantly associated ((β = 0.09 mU/L; 95% CI: 0.03, 0.15; *p* = 0.004), (β = 0.09; 95% CI: 0.03, 0.15; *p* = 0.006), (β = −0.019 mg/L; 95% CI: −0.028, −0.009; *p* = 0.005) and (β = −3.09 mg/L; 95% CI: −6.11, −0.07; *p* = 0.046), respectively).

## 3. Discussion

The main finding of our study was the sex-specific association between rs11804091 and obesity and insulin resistance in girls. We show the association between this SNP and anthropometric, clinical and metabolic obesity-related markers. Moreover, after adjusting for BMI, this SNP remained associated positively with insulin and HOMA-IR, and negatively with QUICKI and adiponectin. Our results suggest that the polymorphism rs11804091, or a flagged variant in LD with it, might have an effect on leptin action with an impact on insulin signalling which does not depend entirely on adiposity.

Leptin and insulin are two body energy sensors that act in the hypothalamus through their respective receptors regulating several peripheral functions. Both promote changes in the expression of hypothalamic neuropeptides to regulate energy balance and glucose metabolism [37]. Two separate studies have shown that the reintroduction of the leptin receptors in the hypothalamus of *LEPR* null mice reduces obesity in different degrees and through different actions [38,39]. When *LEPR* were reintroduced in the pro-opiomelanocortin (POMC) neurones, which usually express *LEPR*, and other hypothalamic regions where *LEPR* expression has been associated with the regulation of food intake, the animals showed a discrete reduction in body weight and adiposity due to an increase in energy expenditure and also an improvement in the glucose and lipid metabolisms [38]. Interestingly, only male mice showed decreased body weight and adiposity in that study, suggesting sex-dependant changes in *LEPR* energy balance regulatory metabolism. Similar results were observed in another study in which *LEPR* was overexpressed in all POMC neurones. In this study, mice reduced their body weight by changing both food intake (decreased) and energy expenditure (increased); these animals also showed lower plasma insulin and glucose levels [39]. In both studies, the improvement in glucose levels and insulin sensitivity was independent of body weight, suggesting that leptin signalling in POMC neurones has a role in regulating glucose homoeostasis and that this regulatory role is not influenced by adiposity [37]. In fact, it has been described that leptin participates in regulating glucose homeostasis and insulin sensitivity by signalling pathways, which include: Janus kinase (JAK), phosphatidylinositol 3’-kinase (PI3K) and extracellular signal-regulated kinase (ERK) [40]. Therefore, the association found in this study between rs11804091 and obesity in females may be related to a lower expression of *LEPR*, which could derive in insulin resistance through mechanisms such as those explained above; however, expression analyses should be performed to confirm this.

Leptin receptor plays an essential role in the physiological effects of leptin. Although some studies have described very high circulating levels of leptin in carriers of LEPR mutations, others have not [15]. In the present study, we did not observe associations between *LEPR* variants and circulating leptin levels, which suggests that SNPs in *LEPR* are not important regulators of circulating leptin levels.

As previously mentioned, the observed relationship between the SNP rs11804091 could be due to an unknown functional variant flagged by it. Indeed, the functional association of an intronic SNP such as rs11804091 with a disease may arise from different potential mechanisms such as altered miRNA binding sites, or changes in TF-binding sites, either in the region of the characterized variant or in that of a second SNP flagged by the first, or to a flagged missense variant that could have an impact on the protein sequence and its functionality. The fact that we did not observe a high LD between this SNP and the other analysed variants indicates that the functional SNP could be at any position in the genome sequence, not necessarily near to the candidate gene. Similarly, we could not define a functional role of rs11804091 affecting miRNA or TF binding sites, since the search of the available databases did not retrieve significant findings.

Among the studied SNP in the present work, we demonstrated the association of five variants (rs11208659, rs11804091, rs10157275, rs9436303 and rs1627238) with obesity in Spanish children and adolescents, from which only the variant rs11208659 had been previously associated with severe early-onset obesity in European children [13], and the rest of the associations are described for the first time. We found no association between other previously described functional *LEPR* SNPs and obesity such as rs1137101 (Gln223Arg), which has been associated with lower and higher obesity risk in Spanish adults [16] and girls [29], respectively, but not in Turkish [24], Polish [25,30], Mexican Mestizo [26] or European [27] children and adolescents, as well as with type 2 diabetes [31]. Another variant, the rs1137100 (Lys109Arg), has also been associated with obesity in European [27] and Indian [28], but not in Mexican Mestizo [26], Spanish [29] or Danish [41] children. Finally, the SNP rs8179183 has been associated with obesity in Mexican Mestizo [26] but not in Spanish [29], Polish [30] or European [27] children. The results of these studies are inconclusive and controversial and may be due to the different genetic background of the populations, as well as to the small sample sizes used.

Our study has several strengths and limitations, which should be mentioned. The main strengths are the high quantity of analysed biomarkers and the strict SNPs’ selection method. The limitations include a relatively small sample size for a genetic association study, which requires further validation in independent and larger populations; and the fact that information on food intake and energy expenditure, whose effects are regulated by leptin, was not available.

In conclusion, we demonstrate for the first time the gender-specific association between rs11804091 and obesity in Spanish girls. Our findings show that this polymorphism is also associated with insulin resistance independently of obesity in girls, suggesting that it might have a potential effect or flag a functional polymorphism that has an effect on the action of leptin on insulin metabolism. It will be valuable to replicate these findings in larger populations to validate the results obtained in the present study.

## 4. Materials and Methods

### 4.1. Study Design

In the present case-control multicentre study, 522 children were recruited, 286 classified as obese (146 boys and 140 girls) and 236 as normal weight (133 boys and 103 girls) according to BMI, using the sex- and age-specific cut-off points published by Cole et al. [42]. The children, aged 6–15 years, were recruited in two Spanish cities (Cordoba and Santiago de Compostela) at primary care centres and schools. Inclusion criteria were European-Caucasian heritage and absence of congenital metabolic diseases. Exclusion criteria were non-European Caucasian heritage, the presence of congenital metabolic diseases (e.g., diabetes or hyperlipidaemia), undernutrition, and the use of any medication to control BP and glucose or lipid metabolism. There were no siblings included in the study. After the initial assessment, parents of children that fulfilled the inclusion criteria were invited to take the children to the paediatric unit of the participating hospitals for a clinical examination. The study aims and procedures were fully explained to parents or guardians prior to written consent been taken, and the children gave their assent.

This study was compliant with the Declaration of Helsinki 1975, revised in 2008, and followed the recommendations of the Good Clinical Practice of the CEE (Document 111/3976/88 July 1990), and the legally enforced Spanish regulation, which regulates the clinical investigation of human beings (RD 223/04 about clinical trials). The Ethics Committee of the Reina Sofía University Hospital of Cordoba, the Ethics Committee on Human Research of the University of Granada and the Bioethics Committee of the University of Santiago de Compostela approved the study (Project identification codes P06-CTS-2203 (04/05/2007) and PI 051968 (25/12/2005).

### 4.2. Anthropometric and Biochemical Measurements

The anthropometric measurements were taken with the children barefooted and in their underwear. A standard beam balance was used to determine body weight (kg), a precision stadiometer was used to measure height (cm) and for WC, with the child standing, an inelastic tape was applied horizontally midway between the lowest rib margin and the iliac crest at the end of a gentle exhalation. BMI was calculated and the *z*-BMI was obtained based on the Spanish references [43]. BP was measured using a mercury sphygmomanometer with an appropriate cuff to the size of the child’s upper right arm and following international recommendations [44]. Blood samples were taken after an overnight fast and clinical biochemical analyses were performed at the laboratories of the participating hospital following internationally accepted quality control protocols. Insulin resistance was evaluated with the HOMA-IR index [45].

### 4.3. Inflammation and Cardiovascular Risk Biomarkers

Inflammation and CVD risk biomarkers were measured on a Luminex^®^ 200™ System (Luminex Corporation, Austin, TX, USA), using three different LINCOplex™ kits of human monoclonal antibodies (Linco Research, St Charles, MO, USA) as previously described [46]. Kit HADK1-61K-A was used to determine adiponectin (CV: 7.9%) and active PAI-1 (aPAI-1) (CV: 6.6%). Kit HADK2-61K-B was used for IL-6 (CV: 7.8%), IL-8 (CV: 7.9%), TNF-α (CV: 7.8%), and leptin (CV: 7.9%), while kit HCVD1-67AK was used for sICAM-1 (CV: 7.9%), sE-selectin (CV: 11.2%), total PAI-1 (tPAI-1) (CV: 6.6%), MPO (CV: 12.3%), and MMP-9 (CV: 6.8%). C-RP (CV: 4%) was determined using a particle-enhanced turbidimetric immunoassay (Dade Behring Inc., Deerfield, IL, USA).

### 4.4. DNA Isolation and Genotyping

Twenty-eight SNPs in the *LEPR* gene were selected from the HapMap and NCBI databases among those with a minor allele frequency (MAF) higher than 0.05 and a minimum pairwise LD of *r^2^* = 0.8 for the Caucasian population, as previously described [46,47]. The QIAamp Blood kit (Qiagen, Valencia, CA, USA) was used to extract genomic DNA from peripheral white blood cells according to the manufacturer’s instructions. Genotyping was performed on 96-well format Sentrix^®^ arrays with the Illumina GoldenGate Assay (Illumina Inc., San Diego, CA, USA).

The genotyping successful rate was >95% for all SNPs. We excluded variants rs4468199 and rs3762274 from the study due to deviation from Hardy-Weinberg equilibrium in the normal-BMI group (*p* < 0.05).

### 4.5. Statistical Analysis

All continuous variables were expressed as mean ± standard error of the mean (SEM). Cholesterol, insulin, HOMA-IR, MMP-9, and tPAI-1 were normalised using log-transformation. Comparisons between normal-BMI and children with obesity variables were assessed using the Student’s _t_-test for unpaired samples. The genotypic relative risk was assessed using logistic regression analysis under an additive model with Bonferroni correction comparing the obese and the normal-BMI group in the whole population and separately by sex. Linear or logistic regression analyses were performed to estimate the association of each SNP with parameters linked to obesity and inflammation and CVD risk biomarkers. Significance was considered at the level of _p_ < 0.05. The statistical analyses were performed with IBM-SPSS Statistics 20 (Armonk, NY, USA) and PLINK version 1.07 [48].

## Figures and Tables

**Table 1:** Anthropometric, clinical, and biochemical parameters of the studied children.

	Normal-BMI	Obese	*p*
*n*	236	286	
Anthropometry			
Sex (M/F)	133/103	146/140	0.252
Age (y)	9.72 ± 0.16	9.43 ± 0.15	0.188
Tanner Stage (Prepuber/Puber)			
Male	113/20	122/24	
Female	83/20	108/32	
Weight (kg)	32.9 ± 0.7	55.9 ± 1.0	<0.001
Height (m)	1.37 ± 0.01	1.41 ± 0.01	0.002
BMI (kg/m^2^)	17.14 ± 0.13	27.59 ± 0.24	<0.001
BMI *z-Score*	−0.17 ± 0.04	3.50 ± 0.08	<0.001
Waist circumference (cm)	60.3 ± 0.5	84.0 ± 0.9	<0.001
Clinical and Metabolic Biomarkers			
Systolic BP (mm Hg)	98 ± 1	111 ± 1	<0.001
Diastolic BP (mm Hg)	60 ± 1	69 ± 1	<0.001
Glucose (mg/dL)	84 ± 1	85 ± 1	0.816
Insulin (mU/L)	5.89 ± 0.23	11.53 ± 0.52	<0.001
HOMA-IR	1.26 ± 0.05	2.45 ± 0.12	<0.001
QUICKI	0.383 ± 0.003	0.347 ± 0.002	<0.001
Triacylglycerols (mg/dL)	55 ± 1	75 ± 2	<0.001
Apo-AI (mg/dL)	149 ± 2	132 ± 2	<0.001
Apo-B (mg/dL)	67 ± 1	71 ± 1	0.006
Cholesterol (mg/dL)	171 ± 2	165 ± 2	0.024
HDL-c (mg/dL)	64 ± 1	51 ± 1	<0.001
LDL-c (mg/dL)	94 ± 2	97 ± 2	0.136
AST (U/L)	23.70 ± 0.48	21.23 ± 0.40	<0.001
ALT (U/L)	16.80 ± 0.57	20.88 ± 0.51	<0.001
GGT (U/L)	8.44 ± 0.27	10.90 ± 0.30	<0.001
Adiponectin (mg/L)	28.23 ± 0.77	22.53 ± 0.66	<0.001
Resistin (μg/L)	9.67 ± 0.34	11.77 ± 0.35	<0.001
Leptin (μg/L)	4.30 ± 0.26	23.15 ± 0.87	<0.001
Inflammation Biomarkers			
C-reactive protein (mg/L)	0.97 ± 0.23	3.44 ± 0.25	<0.001
Interleukin 6 (ng/L)	4.55 ± 0.54	7.03 ± 0.76	0.008
Interleukin 8 (ng/L)	1.57 ± 0.11	2.17 ± 0.15	0.002
TNF-α (ng/L)	3.04 ± 0.11	4.00 ± 0.13	<0.001
Cardiovascular Disease Risk Biomarkers			
MMP-9 (μg/L)	79.72 ± 3.17	87.98 ± 3.92	0.714
MPO (μg/L)	13.18 ± 1.18	21.70 ±1.73	<0.001
sE selectin (μg/L)	22.91 ± 0.77	31.36 ± 1.06	<0.001
sICAM-1 (mg/L)	0.153 ± 0.004	0.174 ± 0.005	<0.001
Active PAI-1 (μg/L)	5.07 ± 0.26	11.92 ± 0.58	<0.001
Total PAI-1 (μg/L)	18.82 ± 0.85	27.11 ± 1.12	<0.001

Mean ± standard error of the mean (SEM). M: male; F: female; y: year; BMI: body mass index; BP: blood pressure; HOMA-IR: homeostasis model assessment for insulin resistance; QUICKI: quantitative insulin sensitivity check index; Apo: apolipoprotein; HDL-c: high-density lipoprotein cholesterol; LDL-c: low-density lipoprotein cholesterol; ALT: alanine transaminase; AST: aspartate transaminase; GGT: gamma-glutamyl transpeptidase; TNF-α: tumour necrosis factor alpha; MMP-9: metalloproteinase-9; MPO: myeloperoxidase; sICAM-1: soluble intracellular adhesion molecule-1, PAI-1: plasminogen activator inhibitor.

**Table 2:** Genotypic distributions of the *LEPR* analysed polymorphisms and its association with obesity in children.

Polymorphism	Function	Allele 1/Allele 2	Case			Control			Minor Allele	Minor Allele		OR (95% CI)	*p*	*p* ^a^
			11	12	22	11	12	22		Case	Control			
**rs11208659**	Intron	**T/C**	**240**	**44**	**2**	**173**	**61**	**2**	**C**	**0.084**	**0.138**	**0.54 (0.35–0.81)**	**0.003**	**0.076**
**rs11804091**	Intron	**A/G**	**193**	**76**	**8**	**181**	**46**	**2**	**G**	**0.166**	**0.109**	**1.64 (1.13–2.39)**	**0.010**	**0.251**
**rs10157275**	Intron	**C/T**	**195**	**83**	**8**	**184**	**46**	**6**	**T**	**0.173**	**0.123**	**1.53 (1.08–2.18)**	**0.017**	**0.444**
**rs9436303**	Intron	**A/G**	**162**	**100**	**24**	**152**	**73**	**11**	**G**	**0.259**	**0.201**	**1.36 (1.02–1.81)**	**0.036**	**0.926**
**rs1627238**	Intron	**C/T**	**186**	**87**	**13**	**167**	**60**	**5**	**T**	**0.198**	**0.151**	**1.40 (1.01–1.94)**	**0.046**	**1**
rs17412175	Intron	T/A	82	146	58	57	120	58	A	0.458	0.502	0.82 (0.64–1.06)	0.133	1
rs9436739	Intron	T/A	231	53	2	178	56	2	A	0.100	0.127	0.74 (0.50–1.10)	0.135	1
rs1137101	Gln223Arg	A/G	85	135	65	76	117	41	G	0.465	0.425	1.16 (0.91–1.48)	0.243	1
rs6673591	Intron	A/G	83	129	74	55	121	60	G	0.484	0.511	0.89 (0.70–1.13)	0.325	1
rs17412723	Intron	A/G	71	155	60	58	114	62	G	0.481	0.509	0.88 (0.68–1.14)	0.329	1
rs6697315	Intron	T/C	126	125	35	92	113	30	C	0.341	0.368	0.88 (0.68–1.14)	0.329	1
rs6704167	Intron	A/T	92	138	56	68	114	52	T	0.437	0.466	0.88 (0.69–1.13)	0.330	1
rs8179183	Lys656Asn	G/C	195	82	9	153	72	10	C	0.175	0.196	0.87 (0.63–1.19)	0.379	1
rs1327118	PRO	G/C	73	143	59	62	121	42	C	0.475	0.456	1.10 (0.85–1.43)	0.472	1
rs1137100	Lys109Arg	A/G	155	112	19	133	89	14	G	0.262	0.248	1.09 (0.82–1.45)	0.552	1
rs3806318	PRO	A/G	158	104	24	116	101	16	G	0.266	0.285	0.92 (0.70–1.21)	0.562	1
rs970468	Intron	T/G	123	135	28	96	113	26	G	0.334	0.351	0.93 (0.71–1.22)	0.588	1
rs3790429	Intron	A/T	187	93	5	161	64	8	T	0.181	0.172	1.09 (0.78–1.51)	0.630	1
rs9436740	Intron	A/T	143	115	24	119	89	26	T	0.289	0.301	0.95 (0.73–1.24)	0.704	1
rs1475397	Intron	C/T	149	118	19	122	95	19	T	0.273	0.282	0.95 (0.72–1.25)	0.712	1
rs11585329	Intron	T/G	215	64	7	171	61	4	T	0.136	0.146	0.94 (0.66–1.33)	0.718	1
rs4655802	Intron	A/G	92	133	55	74	111	41	G	0.434	0.427	1.03 (0.80–1.32)	0.828	1
rs6678033	Intron	G/A	107	136	43	91	111	34	A	0.388	0.379	1.03 (0.80–1.33)	0.829	1
rs6672331	Intron	G/C	273	13	0	224	12	0	C	0.023	0.025	0.94 (0.42–2.12)	0.886	1
rs1137099	Thr85Ala	A/	286	0	0	236	0	0		0	0	-	-	-
rs13306526	Ile503Val	A/	286	0	0	236	0	0		0	0	-	-	-

CI: confidence interval; OR: odds ratio; PRO: promoter. OR adjusted for age, sex and Tanner stage under the additive model. ^a^ *p* values after Bonferroni correction. The bold is the statistic significance for the rows where *p* is lower than 0.05.

**Table 3:** Genotypic distributions of the significant *LEPR* polymorphisms and its association with obesity by sex in children.

Polymorphism	Allele 1/Allele 2	Case			Control			Minor Allele	Minor Allele		OR (95% CI)	*p*	*p* ^a^
		11	12	22	11	12	22		Case	Control			
Females													
rs11208659	T/C	113	26	1	72	3	1	C	0.100	0.155	0.56 (0.31–1.00)	0.050	1
**rs11804091**	**A/G**	**87**	**42**	**4**	**82**	**19**	**0**	**G**	**0.188**	**0.094**	**2.73 (1.47–5.08)**	**0.001**	**0.038**
**rs10157275**	**C/T**	**99**	**37**	**4**	**84**	**17**	**2**	**T**	**0.161**	**0.102**	**1.77 (1.01–3.12)**	**0.045**	1
rs9436303	A/G	77	52	11	68	30	5	G	0.264	0.194	1.49 (0.95–2.32)	0.080	1
rs1627238	C/T	93	41	6	75	23	2	T	0.189	0.135	1.59 (0.95–2.32)	0.079	1
rs1327118	G/C	32	73	30	29	54	12	C	0.493	0.411	1.53 (1.01–2.32)	0.048	1
Males													
**rs11208659**	**T/C**	**127**	**18**	**1**	**101**	**31**	**1**	**C**	**0.068**	**0.124**	**0.50 (0.27–0.92)**	**0.026**	0.681
rs11804091	A/G	106	34	4	99	27	2	G	0.146	0.121	1.23 (0.76–2.02)	0.837	1
rs10157275	C/T	96	46	4	100	29	4	T	0.185	0.139	1.40 (0.89–2.21)	0.147	1
rs9436303	A/G	85	48	13	84	43	6	G	0.253	0.207	1.28 (0.87–1.87)	0.218	1
rs1627238	C/T	93	46	7	92	37	3	T	0.206	0.163	1.33 (0.86–2.04)	0.202	1
rs1327118	G/C	41	70	29	33	67	30	C	0.457	0.489	0.88(0.63–1.24)	0.465	1

CI: confidence interval; OR: odds ratio. OR adjusted for age and Tanner stage under the additive model. ^a^ *p* values after Bonferroni correction. The bold is the statistic significance for the rows where *p* is lower than 0.05.

**Table 4:** Association of rs11804091 with anthropometric, clinical, inflammation and CVD risk biomarkers in girls.

Biomarkers	AA	AG	GG	β (95% CI)	*p*	*p* ^a^
*n*	169	61	4			
Anthropometry						
Height (m)	1.37 ± 0.01	1.41 ± 0.02	1.35 ± 0.05	−0.014 (−0.025, −0.004)	0.010	--
Weight (kg)	43.1 ± 1.4	49.9 ± 2.5	50.5 ± 7.9	6.1 (2.6, 9.6)	0.001	--
BMI (kg/m^2^)	22.44 ± 0.47	24.41 ± 0.78	27.33 ± 2.15	2.20 (0.70, 3.70)	0.004	--
BMI *z-Score*	1.57 ± 0.15	2.08 ± 0.23	3.51 ± 0.45	0.70 (0.22, 1.18)	0.004	--
Waist circumference (cm)	71.56 ± 1.31	76.07 ± 2.14	80.75 ± 2.39	5.46 (2.62, 9.59)	0.055	0.667
Clinical and Metabolic Biomarkers						
Systolic BP (mm Hg)	104 ± 1	108 ± 2	120 ± 3	5.56 (1.97, 9.14)	0.003	0.092
Diastolic BP (mm Hg)	65 ± 1	66 ± 1	73 ± 6	2.49 (−0.51, 5.49)	0.105	0.569
Glucose (mg/dL)	84 ± 1	85 ± 1	79 ± 2	−0.08 (−1.94, 1.78)	0.934	0.989
Insulin (mU/L)	9.11 ± 0.60	12.64 ± 1.15	10.95 ± 3.25	0.14 (0.07, 0.21)	0.0001	0.004
HOMA-IR	1.91 ± 0.13	2.70 ± 0.28	2.17 ± 0.69	0.14 (0.07, 0.22)	0.0002	0.006
QUICKI	0.367 ± 0.003	0.342 ± 0.004	0.349 ± 0.014	−0.019 (−0.028, −0.009)	0.0001	0.005
Triacylglycerols (mg/dL)	69 ± 3	70 ± 4	130 ± 32	8.50 (−0.17, 17.17)	0.056	0.376
Apo-AI (mg/dL)	139 ± 2	131 ± 3	122 ± 9	−8.19 (−15.01, −1.37)	0.019	0.129
Cholesterol (mg/dL)	168 ± 2	169 ± 4	180 ± 14	2.01 (−5.11, 0.55)	0.604	0.334
HDL-c (mg/dL)	55 ± 2	52 ± 2	62 ± 17	−1.83 (−5.68, 2.01)	0.350	0.649
Adiponectin (mg/L)	26.67 ± 0.95	20.96 ± 1.48	23.33 ± 2.30	−4.77 (−7.94, −1.60)	0.004	0.046
Leptin (μg/L)	13.66 ± 1.03	19.01 ± 2.13	19.81 ± 3.69	5.10 (0.04, 1.06)	0.006	0.314
Inflammation Biomarkers						
C-reactive protein (mg/L)	2.29 ± 0.41	3.42 ± 0.52	1.65 ± 0.61	0.84 (−0.45, 2.13)	0.202	0.633
IL-6 (ng/L)	6.03 ± 0.89	5.38 ± 0.99	18.16 ± 9.07	0.92 (−1.91, 3.77)	0.522	0.737
IL-8 (ng/L)	1.80 ± 0.13	1.79 ± 0.25	3.63± 1.51	0.21 (−0.26, 0.69)	0.381	0.637
TNF-α (ng/L)	3.26 ± 0.15	3.84 ± 0.31	4.02 ± 0.94	0.55 (0.04, 1.06)	0.035	0.090
Cardiovascular Disease Risk Biomarkers						
MMP-9 (µg/L)	84.18 ± 4.44	78.82 ± 6.59	75.67 ± 13.56	−5.36 (−19.92, 9.11)	0.494	0.489
MPO (µg/L)	17.27 ± 1.47	21.59 ± 4.28	22.05 ± 7.14	3.47 (−2.63, 9.58)	0.266	0.572
sE-Selectin (µg/L)	26.78 ± 1.22	29.67 ± 2.44	21.99 ± 5.74	2.54 (−1.89, 6.97)	0.263	0.518
sICAM-1 (mg/L)	0.164 ± 0.005	0.160 ± 0.009	0.218 ± 0.047	0.002 (−0.016, 0.021)	0.766	0.889
Active PAI-1 (µg/L)	9.51 ± 0.69	9.63 ± 1.12	15.55 ± 6.81	0.89 (−1.47, 3.26)	0.461	0.441
Total PAI-1 (µg/L)	23.75 ± 1.29	23.56 ± 2.54	28.67 ± 9.17	0.62 (−3.98, 5.23)	0.713	0.707

CI: Confidence interval; BMI: body mass index; BP: blood pressure; HOMA-IR: homeostasis model assessment for insulin resistance; QUICKI: quantitative insulin sensitivity check index; HDL-c: high-density lipoprotein cholesterol; IL: interleukin; TNF-α: tumour necrosis factor alpha; MMP-9: matrix metalloproteinase-9; MPO: myeloperoxidase; sICAM-1: soluble intracellular adhesion molecule-1; sE-selectin: soluble endothelial selectin; PAI-1: plasminogen activator inhibitor. β Coefficients represent the change in absolute traits values of each additional risk allele. General linear or logistic models were used to examine associations, *p* adjusted by age and Tanner stage, *p* ^a^ adjusted by age, Tanner stage, and BMI.

## Supplementary Materials

Supplementary materials can be found at www.mdpi.com/1422-0067/18/8/1690/s1.

## Abbreviations

Apo	Apolipoprotein
AST	Aspartate transaminase
BBB	Blood–brain barrier
BMI	Body mass index
BP	Blood pressure
C-RP	C-Reactive protein
CVD	Cardiovascular disease
ERK	Extracellular signal-regulated kinase
GGT	Gamma-glutamyl transpeptidase
HDL-c	High-density lipoprotein-cholesterol
HOMA-IR	Homeostatic model assessment of insulin resistance
IL	Interleukine
JAK	Janus kinase
LD	Linkage disequilibrium
LDL-c	Low-density lipoprotein-cholesterol
LEPR	Leptin receptor
MAF	Minor allele frequency
MMP-9	Metalloproteinase-9
MPO	Myeloperoxidase
PAI-1	Plasminogen activator inhibitor
PI3K	Phosphatidylinositol 3′-kinase
POMC	Pro-opiomelanocortin
QUICKI	Quantitative insulin sensitivity check index
sICAM-1	Soluble intracellular adhesion molecule-1
SNP	Single nucleotide polymorphisms
TAG	Triacylglycerols
TNF-α	Tumour necrosis factor alpha
WC	Waist circumference
*z*-BMI	BMI *z-Score*
