# Supplementary material for: Leptin Receptor Gene Variant rs11804091 Is Associated with BMI and Insulin Resistance in Spanish Female Obese Children: A Case-Control Study
Source: Int J Mol Sci. 2017 Aug 3;18(8):1690. doi: 10.3390/ijms18081690 (PMC5578080; doi:10.3390/ijms18081690)
Supplement: Supplementary file 1 [file ijms-18-01690-s001.pdf]

Supplementary Material

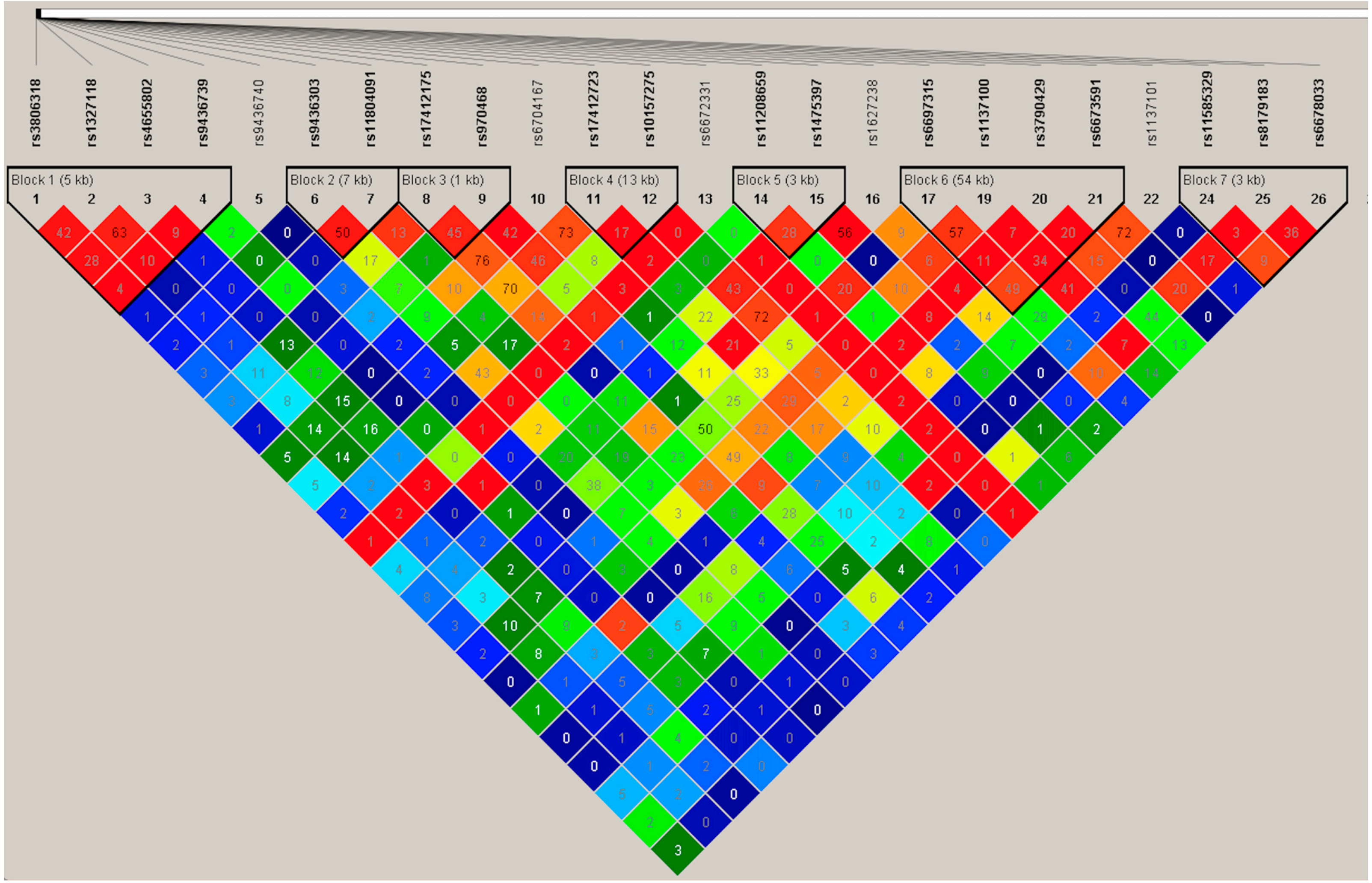

Figure S1. Linkage disequilibrium structure in terms of r^2 of the 26 genotyped *LEPR* polymorphisms in the whole studied population

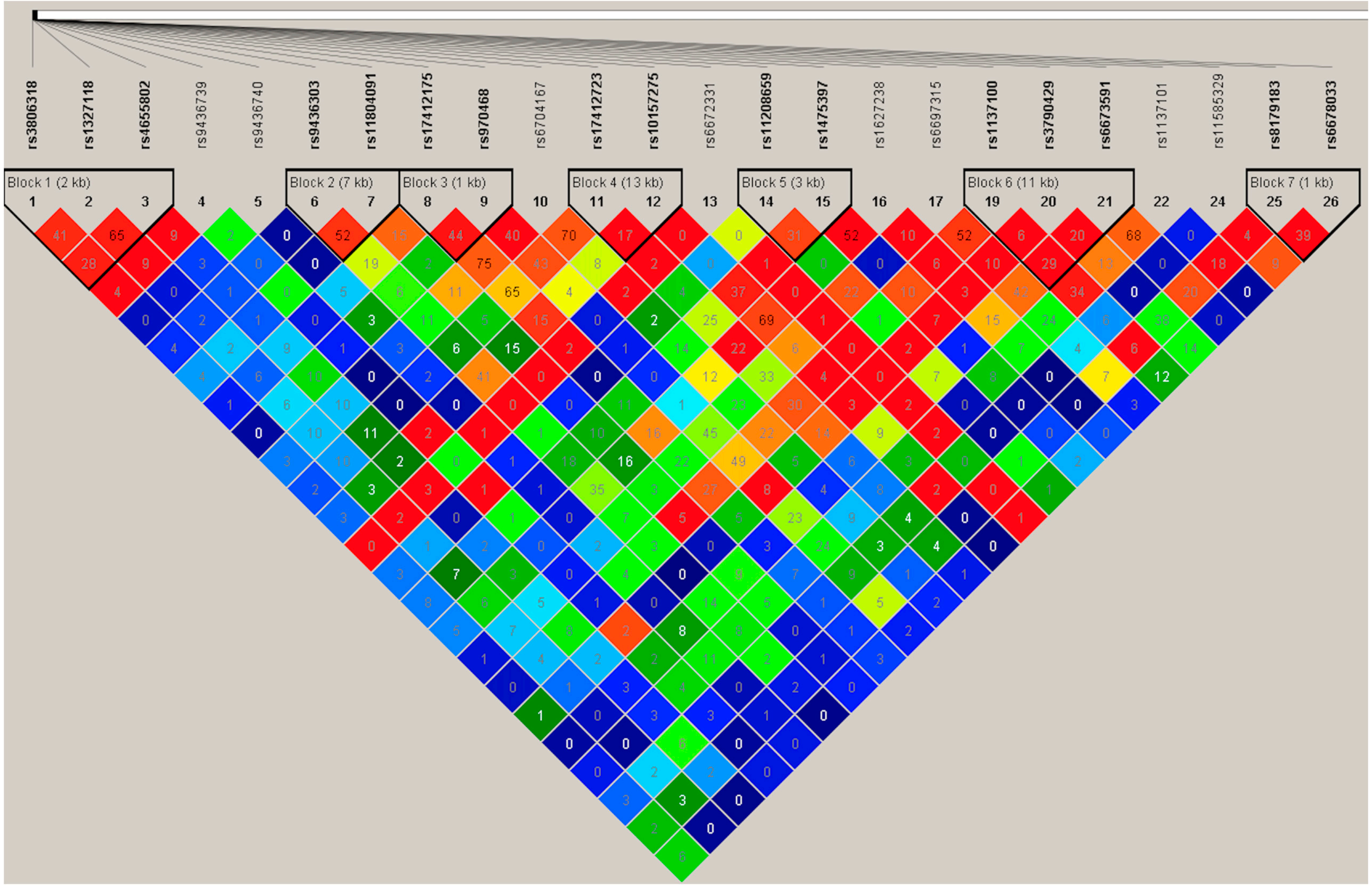

Figure S2. Linkage disequilibrium structure in terms of r^2 of the 26 genotyped *LEPR* polymorphisms in Spanish girls

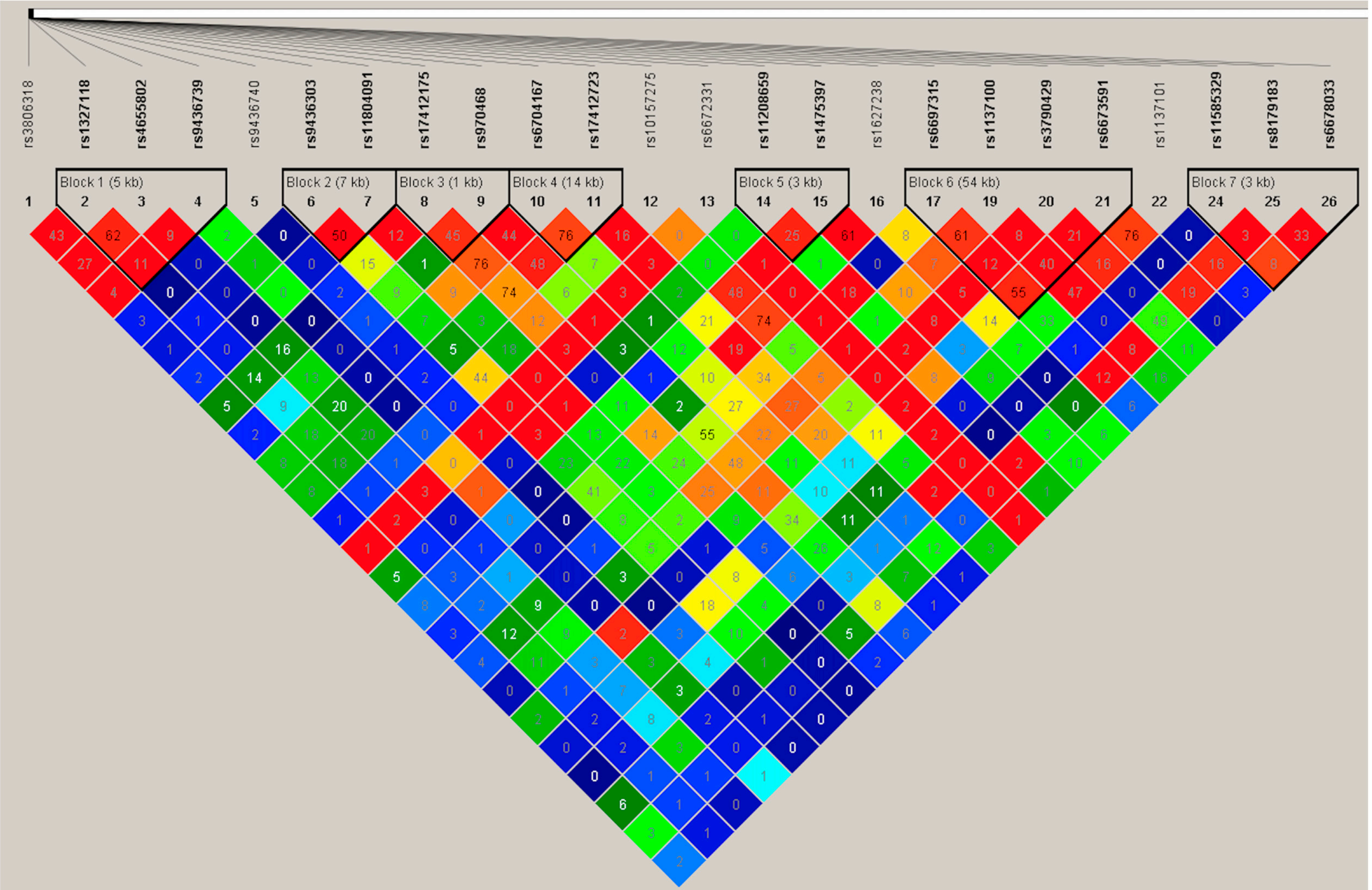

Figure S3. Linkage disequilibrium structure in terms of r^2 of the 26 genotyped *LEPR* polymorphisms in Spanish boys.
